# Sub-nanotesla sensitivity at the nanoscale with a single spin

**DOI:** 10.1093/nsr/nwad100

**Published:** 2023-04-20

**Authors:** Zhiyuan Zhao, Xiangyu Ye, Shaoyi Xu, Pei Yu, Zhiping Yang, Xi Kong, Ya Wang, Tianyu Xie, Fazhan Shi, Jiangfeng Du

**Affiliations:** CAS Key Laboratory of Microscale Magnetic Resonance and School of Physical Sciences, University of Science and Technology of China, Hefei 230026, China; CAS Center for Excellence in Quantum Information and Quantum Physics, University of Science and Technology of China, Hefei 230026, China; CAS Key Laboratory of Microscale Magnetic Resonance and School of Physical Sciences, University of Science and Technology of China, Hefei 230026, China; CAS Center for Excellence in Quantum Information and Quantum Physics, University of Science and Technology of China, Hefei 230026, China; CAS Key Laboratory of Microscale Magnetic Resonance and School of Physical Sciences, University of Science and Technology of China, Hefei 230026, China; CAS Center for Excellence in Quantum Information and Quantum Physics, University of Science and Technology of China, Hefei 230026, China; CAS Key Laboratory of Microscale Magnetic Resonance and School of Physical Sciences, University of Science and Technology of China, Hefei 230026, China; CAS Center for Excellence in Quantum Information and Quantum Physics, University of Science and Technology of China, Hefei 230026, China; CAS Key Laboratory of Microscale Magnetic Resonance and School of Physical Sciences, University of Science and Technology of China, Hefei 230026, China; CAS Center for Excellence in Quantum Information and Quantum Physics, University of Science and Technology of China, Hefei 230026, China; National Laboratory of Solid State Microstructures and Department of Physics, Nanjing University, Nanjing 210093, China; CAS Key Laboratory of Microscale Magnetic Resonance and School of Physical Sciences, University of Science and Technology of China, Hefei 230026, China; CAS Center for Excellence in Quantum Information and Quantum Physics, University of Science and Technology of China, Hefei 230026, China; Hefei National Laboratory, University of Science and Technology of China, Hefei 230088, China; CAS Key Laboratory of Microscale Magnetic Resonance and School of Physical Sciences, University of Science and Technology of China, Hefei 230026, China; CAS Center for Excellence in Quantum Information and Quantum Physics, University of Science and Technology of China, Hefei 230026, China; CAS Key Laboratory of Microscale Magnetic Resonance and School of Physical Sciences, University of Science and Technology of China, Hefei 230026, China; CAS Center for Excellence in Quantum Information and Quantum Physics, University of Science and Technology of China, Hefei 230026, China; Hefei National Laboratory, University of Science and Technology of China, Hefei 230088, China; School of Biomedical Engineering and Suzhou Institute for Advanced Research, University of Science and Technology of China, Suzhou 215123, China; CAS Key Laboratory of Microscale Magnetic Resonance and School of Physical Sciences, University of Science and Technology of China, Hefei 230026, China; CAS Center for Excellence in Quantum Information and Quantum Physics, University of Science and Technology of China, Hefei 230026, China; Hefei National Laboratory, University of Science and Technology of China, Hefei 230088, China

**Keywords:** magnetic sensitivity, spatial resolution, quantum sensing, nanoscale, energy resolution limit

## Abstract

High-sensitivity detection of the microscopic magnetic field is essential in many fields. Good sensitivity and high spatial resolution are mutually contradictory in measurement, which is quantified by the energy resolution limit. Here we report that a sensitivity of 0.5 nT/$\sqrt{\rm Hz}$ at the nanoscale is achieved experimentally by using nitrogen-vacancy defects in diamond with depths of tens of nanometers. The achieved sensitivity is substantially enhanced by integrating with multiple quantum techniques, including real-time-feedback initialization, dynamical decoupling with shaped pulses and repetitive readout via quantum logic. Our magnetic sensors will shed new light on searching new physics beyond the standard model, investigating microscopic magnetic phenomena in condensed matters, and detection of life activities at the sub-cellular scale.

## Teaser

Sub-nanotesla sensitivity at the nanoscale is achieved experimentally using a single spin quantum sensor.

## INTRODUCTION

Along with the advances in science, more investigations are focusing on the microscopic regime, and naturally require the detection of the magnetic field at the microscale [1–11]. For instance, magnetic sensors have been used to search for new interactions beyond the standard model [2,4]. It is necessary to build a microscopic sensor with an extremely high sensitivity for exploring interactions in the short-range regime [3,12]. In condensed matter, observations of some nanoscale magnetic phenomena, such as antiferromagnetic domains with extremely weak magnetization [6] and novel behaviors in mesoscopic superconductors [5,7], all have similar requirements. Biological applications at the microscale, especially for biochemical reactions at the sub-cellular level and nuclear magnetic resonance imaging of a single cell [9], demand both sub-nanotesla sensitivity and sub-micron spatial resolution.

However, the performance of the magnetometers invented so far still needs to be improved for the applications above. As the most sensitive magnetic sensor, superconducting quantum interference devices (SQUIDs) can provide sub-femtotesla sensitivity at the macroscale [13], but working at a smaller scale will dramatically worsen their sensitivity, e.g. tens of nT$\rm /\sqrt{Hz}$ at ∼100 nm [14]. On the other hand, single nitrogen-vacancy (NV) centers in diamond have an extraordinary performance at the nanoscale, but still with a poor sensitivity of tens of nT$\rm /\sqrt{Hz}$ at best [1]. Other (more than 20) kinds of magnetometers have similar behaviors, among which the most prominent are optically pumped magnetometers (OPMs) [15], Bose-Einstein condensates (BECs) [16] and ensemble NV centers [17]. It does not seem realistic to perform high-spatial-resolution detection without impairing the sensitivity. As an empirical limit, the energy resolution limit (ERL) *E_R_* = ℏ is put forward to quantitatively describe the contradiction. Up to now, more than 20 magnetometer technologies comply with the ERL [18].

**Figure 1.**
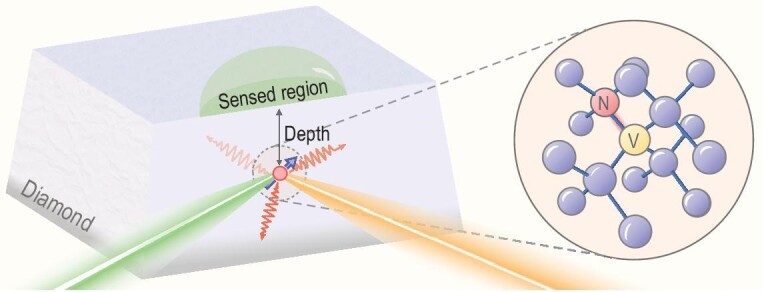
Experimental system: diagram of a single near-surface NV center in diamond. The depth of the NV center determines the dimension of the sensed region. The 532-nm green laser is used for readout of the NV electron spin by collecting fluorescence and mixing charge states, and the 594-nm orange laser is used for readout of the charge state in real-time-feedback initialization. Inset: the atomic structure of the NV center in diamond lattice.

In this work, to overcome the aforementioned problem to some extent, we experimentally achieve a sensitivity of 0.5 $\rm {nT/\sqrt{Hz}}$ for NV centers with depths of more than 30 nm. The NV center is a point defect that consists of a substitutional nitrogen atom and an adjacent vacancy, as shown in Fig. 1, and resides near the diamond surface. The depth is the minimum distance between a sample and an NV center, and thus determines the spatial resolution [1,19]. In our work, the depth of the NV center is measured by detecting the nuclear magnetic resonance from the proton sample put upon the diamond surface [20] (see the Methods section for more details). The detected signal mainly originates from the protons at a distance comparable to the NV depth, which justifies taking the depth roughly as the achieved spatial resolution.

## RESULTS

### Sensitivity optimization

By comprehensively considering multiple experimental limitations, including spin decoherence, initialization and readout errors, and duty cycle, the magnetic sensitivity η_*B*_ for an individual NV center can be accurately formulated as


(1) \begin{eqnarray*}
{\eta _B=\frac{1}{\gamma _e\sqrt{T}}\, \frac{1}{C_T F_r F_i}\, \sqrt{1+\frac{T_{ir}}{T}}},
\end{eqnarray*}

where γ_*e*_ is the gyromagnetic ratio of the electron spin, *T* is the time for phase accumulation and *C_T_* is the remaining spin coherence at *T*. The initialization and readout are both imperfect with *F_i_* and *F_r_* denoting their respective fidelities, and occupy considerable time *T_ir_* leading to a reduced duty cycle. By controlling the temperature stability to the sub-millikelvin level (see Fig. S11 within the online supplementary material), the magnetic field is sufficiently stable so that no calibration of the magnetic field is required during the experiment, and therefore the experimental duty cycle does not need to take into account any other time such as magnetic field calibration. For the near-surface NV center that is exposed to the noise of the diamond surface, the coherence time is shortened dramatically down to tens of microseconds [21], compared with several milliseconds for NV centers deep inside a bulk diamond [22]. For the initialization of the NV center by 532-nm laser illumination, there exist two charge states, of which the useful NV^−^ state occupies ∼70% and the rest is the useless NV^0^ state [23]. The percentage of the NV^−^ state would be further reduced with shallower NV centers [24]. On the optical readout of the NV spin state, the readout fidelity *F_r_* is rather low, ∼3% for typical fluorescence collection efficiencies [1]. Substituting the values above into (1) gives a sensitivity of roughly 0.1 *μ*T$\rm /\sqrt{Hz}$ for typical 10-nm-deep NV centers (if the coherence time is chosen to be 50 $\mu{\rm s}$, *C_T_* = 1/*e*).

Improving the NV spin coherence is of paramount importance. Apart from directly enhancing the sensitivity, long coherence times also enable the use of some time-consuming quantum techniques without dramatically decreasing duty cycles. In order to attenuate the adverse effect from the surface noise, we choose to use NV centers with depths of 10–100 nm combined with the technique of dynamical decoupling [25]. Using an XY16-512 sequence, the coherence time is extended from $146 \pm 5\,\, \mu $s (Hahn echo) to 2.0 ± 0.2 ms for the NV center with a depth of 31.7 ± 1.1 nm. Besides, the magnetic noise from ^13^C nuclear spins in the diamond lattice is removed by ^12^C isotope purification.

**Figure 2.**
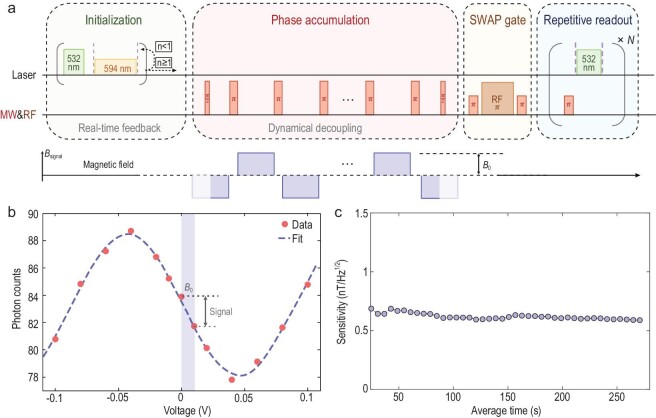
Magnetic field measurement. (a) Experimental sequence comprises initialization, phase accumulation and readout. Real-time feedback is performed to initialize the NV charge state. The magnetic field is measured by encoding it into an accumulated phase using dynamical decoupling sequences. The technique of repetitive readout is employed to improve the readout fidelity. Fluorescence photons are collected during the intervals between two purple dashed lines. Waveform *B*_signal_ is generated by the AWG and fed into a coil to produce a small magnetic field. For further details, see Fig. S4 within the online supplementary material. (b) Interference pattern for magnetic sensing as a function of the AWG voltage. The dashed line is a fitting of the experimental data. (c) Magnetic sensitivity measured as a function of the time of data accumulation in the experiment. The beginning deviation from the final value is due to the small data amount, and the values converge as the data accumulates continuously.

With millisecond-scale spin coherence, two quantum techniques, real-time feedback for NV negative state preparation [23] and repetitive readout via quantum logic [1], can be integrated into the measurement sequence to improve initialization and readout fidelities. Through hundreds of real-time feedback loops (the first block of Fig. 2a), the NV^−^ state can be picked out with a 99% success rate [23,26], but too many loops will degrade the sensitivity. After the sensitivity is optimized, the initialization fidelity of the NV^−^ state is better than 92% (see the Methods section). High-fidelity readout is realized by transferring the NV spin state to the nuclear spin of the adjacent nitrogen atom through a swap gate[27,28], then followed by thousands of readouts of the nuclear spin (the last two blocks of Fig. 2a). The nondestructive nature of the nuclear spin after every readout is ensured by a high magnetic field (7662 G in our setup). The number of readout cycles is also optimized, and executing 2500 cycles (1.44 ms) gives a fidelity of roughly 84%.

### Magnetic field sensing and sensitivity

**Figure 3.**
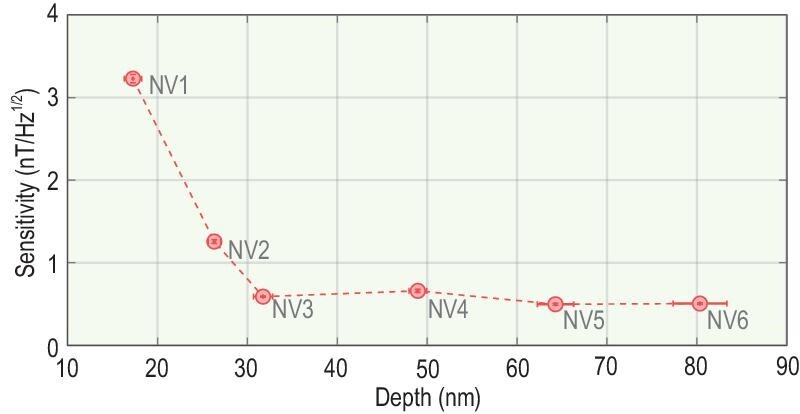
Measured magnetic field sensitivities for six near-surface NV centers with different depths below the diamond surface.

The interference sequence for magnetic field measurement is plotted in Fig. 2a, composed of initialization, phase accumulation and readout. The dynamical decoupling sequences in the second block are used to encode the detected magnetic field into the phase of the NV spin. It is worth noting that the use of high-frequency microwaves (MWs) and the existence of the ^15^N hyperfine coupling make it rather difficult to apply strong π/2 and π pulses for the NV electron spin. Therefore, shaped π/2 and π pulses optimized by the gradient ascent pulse engineering (GRAPE) algorithm [29] are adopted here for a lower amplitude and a higher fidelity (see the Methods section for more details). The detected magnetic field is produced by a copper coil into which a waveform output from an arbitrary waveform generator (AWG) is fed. Changing the AWG voltage varies the number of collected photons in Fig. 2b. The detected magnetic fields are determined with coefficient ≈112 nT/V, which is obtained by fitting the interference pattern. With a specific magnetic field applied, the sensitivity of our system is given by the asymptotic behavior in Fig. 2c, after averaging the data for several hundred seconds (see the Methods section). To find how the sensitivity is affected by the noise from the diamond surface, NV centers with different depths are investigated. By repeating the established procedures above, the sensitivities for six NV centers are measured and displayed in Fig. 3. The sensitivity is improved gradually as the NV depth increases, and saturates at about 0.5 $\rm nT/\sqrt{Hz}$. This implies that the effect of the surface noise is no longer the major limiting factor when the depth reaches ∼30 nm. Note that the sensitivity of 0.5 $\rm nT/\sqrt{Hz}$ is the highest record for a single-NV center at room temperature.

### Energy resolution

**Figure 4.**
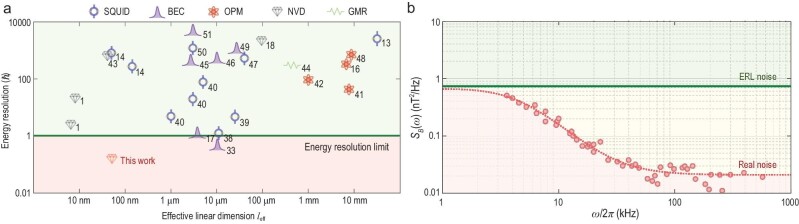
Energy resolution per bandwidth and the measured noise spectrum. (a) Reported energy resolution per bandwidth *E_R_* for different magnetometers versus their effective linear dimensions *l*_eff_. Line shows $E_R\equiv {\langle \delta B^2\rangle Tl^3_{\rm {eff}}}/{(2\mu _0)}=\hbar$. The energy resolution of NV3 in Fig. 3 is labeled ‘This work’. SQUID, superconducting quantum interference device; BEC, Bose-Einstein condensate; OPM, optically pumped magnetometer; NVD, nitrogen-vacancy center in diamond; GMR, giant magnetoresistance. For further details, see the reference citations indicated by numbers in (a) and Table S1 within the online supplementary material. (b) Noise spectral density of the same NV in (a) measured under multiple dynamical decoupling sequences. The red dashed line is the Lorentzian fit. The noise level constrained by *E_R_* = ℏ is indicated by the green solid line (ERL noise). The noise densities for frequencies greater than 100 kHz are below the ERL noise.

In the following, the ERL is adopted to benchmark the NV sensors constructed above. As discussed in the literature [19], the ERL quantitatively depicts the contradiction between the spatial resolution and the sensitivity. Among the more than 20 kinds of magnetometers, SQUID, BEC, OPM and NV centers have demonstrated energy resolution near one ℏ [19], and all of them have practical importance. Thus, these four types of magnetometers are mainly included in Fig. 4a for comparison. The energy resolution combines field, temporal and spatial resolutions in a concise form, which simply reads


(2) \begin{eqnarray*}
E_R\equiv \frac{\langle \delta B^2\rangle T l^3_{\rm {eff}}}{2\mu _0}\ge \hbar ,
\end{eqnarray*}

where *E_R_* is the energy resolution per bandwidth and is greater than Planck’s constant ℏ. In the expression of *E_R_*, μ_0_ is the vacuum permeability, *T* is the measurement time, 〈δ*B*^2^〉 is the magnetic field variance and the sensitivity $\eta _B=\langle \delta B^2\rangle ^{1/2}\sqrt{T}$. The symbol *l*_eff_ represents the effective linear dimension of the sensor and determines the spatial resolution.

The ERL is not actually a universally proven quantum limit, though strictly derived for SQUIDs [30] and spin-precession ensembles [31] under some general assumptions. Recently, a theoretical scheme has been proposed to surpass the ERL by using ferromagnetic torque sensors [12], and an experiment has achieved energy resolution below ℏ with a single-domain Bose–Einstein condensate magnetometer (*E_R_* = 0.48 ℏ and *E_R_* = 0.075 ℏ if the duty cycle of the experiment is not considered) [32]. Additionally, single quantum systems (SQSs), e.g. single-NV centers and trapped ions, also have the potential to achieve energy resolution below ℏ [19]. It is worth noting that the effective linear dimension *l*_eff_ for SQSs is not the size of the system wave function (ultimately limiting the spatial resolution), but the achieved spatial resolution when SQSs are used to detect external objects such as a piece of material in condensed matter physics [7] or a protein in structural biology [1], not internal objects like residual atoms in vacuum [33] or spins in a diamond lattice [11]. In this regard, as analogous to ensemble sensors (e.g. OPM, BEC and NV ensembles), shrinking *l*_eff_ for SQSs always dramatically increases the noise and then shortens coherence times, for which the measurements performed by SQSs are still not below ℏ either [1,19,34].

To find the best energy resolution in our work, by respectively multiplying the spatial resolutions achieved by detecting external nuclear spins, the energy resolutions for six NV centers are obtained (see Table S2 within the online supplementary material). The energy resolution given by the 31.7-nm-deep NV center (NV3 in Fig. 3) is 0.18 ± 0.01 ℏ. As displayed in Fig. 4a, it gives the best energy resolution among the four types of magnetometers (SQUID, BEC, OPM and NV centers).

Essentially, *E_R_* = ℏ gives the noise level at a certain dimension that the magnetic sensor with that dimension must comply with. Therefore, we proceed to investigate the noise felt by the NV center. The noise spectrum [35] for the NV center with the best energy resolution (NV3) is derived from its decoherence behaviors under multiple dynamical decoupling sequences (see the Methods section and Fig. S8 within the online supplementary material). As shown in Fig. 4b, the measured noise spectral densities are all well below those constrained by *E_R_* = ℏ for frequencies above 100 kHz. The best energy resolution (0.18 ℏ in this work) can approach 0.03 ℏ if initialization and readout fidelities as well as duty cycles in the experiments are further improved.

## CONCLUSION

In conclusion, the main drawbacks for near-surface NV centers, such as short coherence times, low-fidelity initialization and low-efficiency readout, are all elegantly overcome by harnessing multiple quantum techniques. The highest sensitivity (0.5 $\rm {nT/\sqrt{Hz}}$) at the nanoscale is experimentally achieved, signifying that our sensor takes the combined advantages of magnetic field, temporal and spatial resolutions over all other magnetometers. Therefore, it opens up a new exploration region on searching new short-range interactions, microscopic magnetic phenomena in condensed matter and detection of life activities at the macro-molecule scale, all demanding both good magnetic sensitivity and high spatial resolution. The best energy resolution 0.18 ± 0.01 ℏ is given by combining the achieved sensitivity and spatial resolution. In the future, it can be further improved by several orders, through reducing the surface noise with surface treatments [1] and suppressing the spin-lattice relaxation with cryogenic temperatures [36].

## METHODS

### Diamond sample and experimental setup

The targeted NV centers are located in a bulk diamond with top and lateral sides perpendicular to the crystal axes [100] and [110], respectively. The top layer with a thickness of several micrometers is grown with an isotopically purified carbon source (99.999% ^12^C) and the nitrogen concentration is less than 5 ppb. The NV centers are created with a density of 5 × 10^−8^ cm^−2^ 15- and 22.5-keV $\rm {^{15}N^+}$-ion implantation in two different regions and followed by annealing in vacuum for 4 h at 1000 °C. The luminescence rates of the NV centers are distributed within 150–230 kcounts/s. The details of the main experimental setup used in this work are similar to our previous work [23]. The diamond is mounted on a typical optically detected magnetic resonance confocal setup and a multichannel pulse blaster is used to synchronize the microwave circuit. The oil objective is used to focus both the 594-nm laser and the 532-nm laser on the NV center. Before entering the objective, all laser beams travel twice through acousto-optic modulators to protect the longitudinal relaxation time and NV^−^ charge state from laser leakage effects. With the use of avalanche photodiodes and a counter card, the fluorescence photons can be recorded. The microwave circuit generates the 18.6- and 24.3-GHz MW pulses for controlling the electron spin, which are combined with radio-frequency (RF) pulses and delivered into the coplanar waveguide. The external magnetic field (≈7662 G) is produced by a permanent magnet. Additionally, the Zurich Instruments AWG includes an integrated counter that enables us to perform real-time feedback for preparing NV^−^. For the overall setup, see Fig. S2 within the online supplementary material.

### Contradictions between multiple techniques and solutions

The techniques used here are contradictory to a certain degree and demand many technical improvements for being combined together. The contradictions between them and the corresponding solutions are graphically represented in Fig. S1 within the online supplementary material and summarized here as follows.

1. If the techniques of charge initialization and repetitive readout are arbitrarily used, due to long times consumed by them, they will not improve the sensitivity but in turn lower it by reducing the duty cycle. In this work, the coherence times for near-surface NV centers are extended to several milliseconds comparable to those consumed by them, and these two techniques are elegantly optimized to largely enhance the sensitivity.
2. The high magnetic field used for repetitive readout poses challenges to the coherent manipulation of the NV spin in the experiments. In this work, it is solved by using low-amplitude and high-fidelity shaped pulses.
3. The coherence time is dramatically shortened when the NV center draws near the diamond surface. In this work, the technique of dynamical decoupling is used to suppress the surface noise and prolong the coherence time.
4. Continuously running experiments without reducing the duty cycle is challenging due to the field instability under a high magnetic field (∼7662 G in this work). In this work, the high magnetic field is stabilized below 0.1 G through active temperature feedback, without the need to frequently calibrate the MW frequency.

### Duty cycle

The duty cycle in our experiments is mainly affected by the initialization and readout time and the time to calibrate the MW frequency. With the techniques of real-time feedback and repetitive readout, both the initialization fidelity and the readout fidelity can be improved above 99%, but it takes a great amount of time and dramatically reduces the experimental duty cycle. The cycle numbers for both processes are therefore optimized to realize the best sensitivity for each NV center. Besides, the high magnetic field used here must be stabilized; otherwise, frequently calibrating the MW frequency during the experiment will reduce the duty cycle.

### Magnetic field stability

In this work, the magnetic field is stabilized below 0.1 G without the need for time-consuming calibration. The most significant factor affecting the magnetic field stability is temperature fluctuation. The temperature disturbs the magnetic field mainly by changing the magnetization of the magnets (0.12$\%\, {}^{\circ }{\rm C}{}^{\text{-1}}$ for NdFeB magnets) and the position of the NV center inside the magnetic field. The temperature fluctuation is reduced to less than 1 mK using an elaborately designed configuration for heat isolation and proper proportional-integral-derivative (PID) parameters for feedback. The latter is more remarkable especially for a field gradient of over 2 G/*μ*m at high magnetic fields (7662 G). Changes in temperature and humidity outside the temperature-controlled box can affect the distance between the sample and the magnet through the optical platform. Figure S9 within the online supplementary material shows the shift of the resonance spectral peaks during the experiments, which shows that the bias field oscillates within ∼0.1 G. The oscillation is caused by the periodic cooling of the air conditioner (through the optical table thermal expansion) affecting the distance between the magnet and the sample. As for the temperature control system, attention should mainly be paid to heat isolation since PID feedback is easily achieved at the best level (only noise is left with no distinct oscillation).

### The system Hamiltonian

The NV electron spin (*S* = 1) in the ground state of the NV^−^ triplet and the adjacent ^15^N nuclear spin (*I* = 1) comprise our nanoscale system. The Hamiltonian with an external magnetic field *B*_0_ ≈ 7662 G applied along the axis of the NV is given by


(3) \begin{eqnarray*}
H_0 \approx \overbrace{D S_z^2+\gamma _e B_0 S_z}^{\rm {NV}}+\overbrace{\gamma _{\rm N} B_0 I_z^{\rm N}+A_\parallel S_z I_z^{\rm N}}^{^{15}\rm {N}},
\end{eqnarray*}

where γ_*e*_ and γ_N_ are the gyromagnetic ratios of the electron spin and the ^15^N nuclear spin, respectively; *S_z_* and $I_z^{\rm N}$ are the components of three spin operators along the axis of the NV. The hyperfine interaction *A*_∥_ is roughly 3.03 MHz. Here *D* ≈ 2870 MHz is the zero-field splitting of the NV ground state. MW and RF with multiple frequencies are imposed to coherently control the electron spin and the ^15^N nuclear spin with the control Hamiltonian


(4) \begin{eqnarray*}
H_c(t) &=& \Omega ^{\rm MW}(t)\cos (\omega ^{\rm MW} t+\phi ^{\rm MW}(t))S_x \nonumber\\
&&+\, \Omega ^{\rm RF}(t)\cos (\omega ^{\rm RF} t+\phi ^{\rm RF}(t))I_x^{\rm N}.\nonumber\\
\end{eqnarray*}

We implemented the quantum circuit (Fig. 2a) by transforming to a suitable interaction picture with some rotation-wave approximations.

### Determination of the NV depth

In our work, the depth of the NV center is obtained by detecting the nuclear magnetic resonance signal from the proton sample put upon the diamond surface. Glycerine or immersion oil (IMMOIL-F30CC) of the objective is placed upon the diamond surface and the fluctuating magnetic field from the ^1^H nuclear spins in the samples is measured using an XY16-N dynamical decoupling pulse sequence. The fluctuating magnetic field causes an extra decoherence of the NV spin, which is described by [20]


(5) \begin{eqnarray*}
C(\tau ) \approx \exp\! \bigg [-\frac{2}{\pi ^{2}} \gamma _{e}^{2} B_{\mathrm{RMS}}^{2} K(N \tau )\bigg ],
\end{eqnarray*}

where γ_*e*_ is the electron gyromagnetic ratio and $B_{\mathrm{RMS}}^{2}$ is the root-mean-square magnetic field “$B_{\mathrm{RMS}}^{2}$ is the *root*-mean-square magnetic field”?"?>produced by the proton spins. Here *K*(*N*τ) depends on the pulse sequence, the coherence time $T_{2n }^*$ and the diffusion coefficient of the proton spins [20]. For a diamond with a [100] top surface, $B_{\mathrm{RMS}}^{2}$ for the NV depth *d*_NV_ is


(6) \begin{eqnarray*}
B_{\mathrm{RMS}}^{2}=\rho \bigg (\frac{\mu _{0} \hbar \gamma _{n}}{4 \pi }\bigg )^{2}\bigg (\frac{5 \pi }{96 d_{\mathrm{NV}}^{3}}\bigg ),
\end{eqnarray*}

where γ_*n*_ is the gyromagnetic ratio of ^1^H spins and ρ is the nuclear spin number density (ρ = 66 nm^−3^ for glycerine by calculation, and ρ = 69.5 nm^−3^ for immersion oil measured with the EDUMR20-015V-I NMR system). The NV depths can be extracted by fitting with the equations above. The results are summarized in Table 1, and more details can be found in Fig. S3 within the online supplementary material.

**Table 1.** The depths of the NV centers used in this experiment.

No.	1	2	3	4	5	6
Depth (nm)	17.3 ± 1.0	26.3 ± 0.7	31.7 ± 1.1	49.0 ± 1.0	64.3 ± 2.0	80.3 ± 3.0

### Measurement of magnetic sensitivity

In order to determine the magnetic sensitivity experimentally, we first have to measure the number of collected photons *N*_ph_ against the AWG voltage *V* proportional to the amplitude of the small magnetic field produced by the coil. The magnetic field magnitude per unit voltage *B_V_* is obtained by fitting with


(7) \begin{eqnarray*}
N_{\rm ph}(v)=a\sin (\gamma _e T B_V V+\phi )+c,
\end{eqnarray*}

where *T* is the interrogation time, γ_*e*_ is the gyromagnetic ratio of the NV spin, and *B_V_, a* and *c* are the fitting parameters. Subsequently, we apply a specific magnetic signal and measure the signal-to-noise ratio (SNR) over a range of experimental times. The sensitivity is then determined by


(8) \begin{eqnarray*}
\text{Sensitivity} = \frac{\text{Signal amplitude}}{\text{SNR per } \sqrt{\text{unit time}}}.
\end{eqnarray*}

The determined sensitivity is approximately equal to that estimated based on (1) (see Table S2 within the online supplementary material). The optimized parameters for six centers are summarized in Table S2, and the corresponding results are displayed in Fig. 2 and Fig. S5 within the online supplementary material.

The method for estimating the sensitivity based on (1) is given here. The fidelity of conventional readout is $F_{R_0}=[{{1+2(\alpha _0+\alpha _1)/(\alpha _0-\alpha _1)^2}}]^{-1/2}$, where α_0_ and α_1_ are the average numbers of collected photons by reading out states |0〉 and | ± 1〉 one time. As for repetitive readout, the fidelity is given by $F_{R_N}=[{{1+2(\alpha _0+\alpha _1)/(N(\alpha _0-\alpha _1)^2)}}]^{-1/2}=[{{1+(1/F_{R_0}^2-1)/N}}]^{-1/2}$, where *N* is the number of readout cycles. The initialization fidelity of the NV^−^ charge state is approximately 92%. The population *p* of the electron state |0〉 is approximately 90%, which affects the final sensitivity by a factor of (3*p* − 1)/2. The interrogation time for sensing a small magnetic signal is determined as the length of the dynamical decoupling sequence after which the coherence remains roughly 0.5–0.6. The relevant experimental parameters of each NV center are gathered in Table S2 within the online supplementary material. Based on (1), the sensitivity of each NV center is estimated with the results listed in Table S2. Overall, the estimation results are consistent with the measured sensitivities. Small deviations may originate, on the one hand, from imperfect implementations of higher-order dynamical decoupling sequences due to MW amplitude fluctuations, and on the other hand, from the difference between the actual coherence (0.5–0.6) and that used for the estimation (fixed as 0.6).

Besides, it is worth noting that the parameters of the dynamical decoupling sequence used by each NV center just give a lower limit to the measurement bandwidth, and the same sensitivity can be achieved by increasing the order of the decoupling sequence for sensing magnetic signals with higher frequencies. These NV centers have a common bandwidth at higher frequencies, and, thus, although the parameters used for each NV center are different, the results can be put together for comparison.

### Noise analysis

The analysis of the noise felt by the NV center is based on the technique of spectral decomposition [35]. Generally, the spin coherence decays as a function of time,


(9) \begin{eqnarray*}
C(t) = \exp ({-\Delta \phi ^2(t)/2}),
\end{eqnarray*}

due to the interactions with surrounding spins. The phase variance Δφ^2^(*t*) depends on the noise spectrum *S*(ω) of the environment and the filter function *F_T_*(ω) of the dynamical decoupling sequence:


(10) \begin{eqnarray*}
\Delta \phi ^2(t)=\gamma _e^2 \int _{0}^{\infty } S(\omega ) {F_{T}(\omega )}\frac{d \omega }{\pi }.
\end{eqnarray*}

The filter function for high-order dynamical decoupling sequences can be approximated as


(11) \begin{eqnarray*}
F_{T}(\omega )\approx 2\pi T\, \frac{4}{\pi ^2}\sum _{k=-\infty }^{\infty }\frac{\delta (\omega -(2k+1)\omega _0)}{(2k+1)^2},
\end{eqnarray*}

where *T* is the total evolution time and ω_0_ = π*N*/*T*. Combining (9), (10) and (11), the noise spectral density at frequency ω_0_ is given by


(12) \begin{eqnarray*}
\frac{8}{\pi ^2}\sum _{k=0}^{\infty }\frac{S((2k+1)\omega _0)}{(2k+1)^2}=\frac{-2\ln C(T)}{\gamma _e^2T}.
\end{eqnarray*}

In order to acquire a wide-range noise spectrum, multiple dynamical decoupling sequences with different orders are executed, as shown in Fig. S8 within the online supplementary material. The effect of spin-lattice relaxation is deducted from the decay curve for millisecond-scale spin coherence. Besides, according to (15) below, we build an iterative formula for a more accurate calculation:


(13) \begin{eqnarray*}
S_{n}(\omega _0)=\frac{\pi ^2}{8}S_0(\omega _0)-\sum _{k=1}^{\infty }\frac{S_{n-1}((2k+1)\omega _0)}{(2k+1)^2} \nonumber\\
\end{eqnarray*}

with *S*_0_(ω_0_) the right-hand side of (15). When the iteration number *n* is large enough, *S_n_*(ω_0_) converges to the noise spectrum *S*(ω_0_) and, generally, *n* = 1 or 2 (*n* = 1 in our case) is enough due to its intrinsic fast convergence. Besides, the noise level constrained by the ERL in Fig. 4 is given by


(14) \begin{eqnarray*}
S(\omega )=\frac{2\mu _0\hbar }{e\, l_{\text{eff}}^3},
\end{eqnarray*}

where the number *e* in the denominator originates from the spin decoherence during phase accumulation.

### Real-time feedback for NV^−^ preparation

The 594-nm orange laser can excite NV^−^ but cannot excite NV^0^ since their zero-phonon lines are 637 and 575 nm, respectively. Therefore, if one photon is recorded during laser illumination, NV^−^ is prepared [23,26]. If not, repeat it until one photon is recorded. The 532-nm laser is turned on to mix charge states before photon counting during each cycle. To evade the reduction of the duty cycle, the experimental parameters are carefully optimized: 970 ns for state readout, 95 ns for state mixing and 100 cycles for the upper bound of feedback loops. The results are shown in Fig. S7 within the online supplementary material.

### Optimal control

The high magnetic field (≈7662 G) poses challenges to the coherent manipulation of the NV spin in the experiment. The existence of the ^15^N hyperfine coupling demands rather strong MW pulses for simultaneous manipulation of the NV spin in two subspaces of the ^15^N spin states. However, the use of high-frequency MWs in a high magnetic field, due to the great attenuation loss in the circuits, makes it difficult to achieve such strong MW pulses. To achieve high-fidelity manipulation with fairly weak MW pulses, the technique of optimal control is employed in dynamical decoupling sequences, and the shaped pulses are optimized numerically by the GRAPE algorithm detailed in Fig. S6 within the online supplementary material.

### Signal of a proton

The magnetic signal of a single proton located right above the NV with a depth of *d*_NV_ is given by


(15) \begin{eqnarray*}
\eta _p=\frac{\mu _0\hbar \gamma _p}{4\pi }\, \frac{1}{{d_{\mathrm{NV}}}^3},
\end{eqnarray*}

where γ_*p*_ is the gyromagnetic ratio of the proton spin and μ_0_ is the vacuum permeability. The sensitivity needed for detecting a single proton (SNR of 1) with 1 s (100 s) of data accumulation is calculated as a function of NV depth, which is plotted as a blue solid (green dashed) line in Fig. S10 within the online supplementary material. The minimum number of detectable proton nuclear spins is 6.7 for the NV with a depth of 31.7 nm (data accumulation for 1 s, SNR = 1).

## Supplementary Material

nwad100_Supplemental_File
