# Supplementary material for: Sub-nanotesla sensitivity at the nanoscale with a single spin
Source: Natl Sci Rev. 2023 Apr 20;10(12):nwad100. doi: 10.1093/nsr/nwad100 (PMC10632795; doi:10.1093/nsr/nwad100)
Supplement: nwad100_Supplemental_File [file nwad100_supplemental_file.pdf]

# Supplemental Material for
# Sub-nanotesla Sensitivity at the Nanoscale with a Single Spin

Table S1. **A summary of energy resolution per bandwidth for different magnetometers.** For single NV centers, the relation between the effective linear dimension $l_{\text{eff}}$ and the NV depth $d_{\text{NV}}$ is given by $l_{\text{eff}}^3 = \frac{4\pi}{3} \cdot d_{\text{NV}}^3$.[1]

| № | Type | $l_{\text{eff}}$ (m) | $\eta_B$ (T/$\sqrt{\text{Hz}}$) | Ref. | $E_R(\hbar)$ |
|---|---|---|---|---|---|
| 1 | NV | $6.4 \times 10^{-09}$ | $5.3 \times 10^{-08}$ | 1 | 2.84 |
| 2 | BEC | $1.1 \times 10^{-05}$ | $5.0 \times 10^{-13}$ | 17 | 1.24 |
| 3 | SQUID | $3.7 \times 10^{-06}$ | $2.6 \times 10^{-12}$ | 38 | 1.34 |
| 4 | SQUID | $2.5 \times 10^{-05}$ | $2.8 \times 10^{-13}$ | 39 | 4.68 |
| 5 | SQUID | $1.0 \times 10^{-06}$ | $3.6 \times 10^{-11}$ | 40 | 4.89 |
| 6 | NV | $8.1 \times 10^{-09}$ | $1.1 \times 10^{-07}$ | 1 | 23.9 |
| 7 | SQUID | $3.0 \times 10^{-06}$ | $1.4 \times 10^{-11}$ | 40 | 20.0 |
| 8 | OPM | $7.6 \times 10^{-03}$ | $1.6 \times 10^{-16}$ | 41 | 43.5 |
| 9 | SQUID | $5.0 \times 10^{-06}$ | $1.3 \times 10^{-11}$ | 40 | 79.7 |
| 10 | OPM | $1.0 \times 10^{-03}$ | $5.0 \times 10^{-15}$ | 42 | 94.3 |
| 11 | NV | $4.0 \times 10^{-08}$ | $5.6 \times 10^{-08}$ | 43 | 774 |
| 12 | SQUID | $1.4 \times 10^{-07}$ | $5.1 \times 10^{-09}$ | 14 | 278 |
| 13 | GMR | $4.3 \times 10^{-04}$ | $3.2 \times 10^{-14}$ | 44 | 320 |
| 14 | OPM | $6.6 \times 10^{-03}$ | $5.4 \times 10^{-16}$ | 16 | 330 |
| 15 | BEC | $2.7 \times 10^{-06}$ | $7.7 \times 10^{-11}$ | 45 | 447 |
| 16 | BEC | $1.0 \times 10^{-05}$ | $1.2 \times 10^{-11}$ | 46 | 543 |
| 17 | SQUID | $4.0 \times 10^{-05}$ | $1.5 \times 10^{-12}$ | 47 | 543 |
| 18 | OPM | $8.7 \times 10^{-03}$ | $5.4 \times 10^{-16}$ | 48 | 726 |
| 19 | SQUID | $5.0 \times 10^{-08}$ | $4.2 \times 10^{-08}$ | 14 | 832 |
| 20 | BEC | $2.7 \times 10^{-05}$ | $3.9 \times 10^{-12}$ | 49 | 1,148 |
| 21 | SQUID | $3.0 \times 10^{-06}$ | $1.1 \times 10^{-10}$ | 50 | 1,233 |
| 22 | NV | $9.4 \times 10^{-05}$ | $9.0 \times 10^{-13}$ | 18 | 2,598 |
| 23 | SQUID | $3.1 \times 10^{-02}$ | $1.5 \times 10^{-16}$ | 13 | 2,644 |
| 24 | BEC | $3.0 \times 10^{-06}$ | $2.3 \times 10^{-10}$ | 51 | 5,389 |
| 25 | BEC | $1.0 \times 10^{-05}$ | $3.4 \times 10^{-13}$ | 33 | 0.48 |

[1] Supplementary Reference 1: Mitchell MW and Alvarez SP. *Colloquium*: Quantum limits to the energy

Table S2. **Experimental parameters and results of our system.** "Total time" means the total time of a single experiment, including initialiation time, interrogation time, and readout time. $\eta_t$ is the sensitivity calculated based on Eq.1 and $\eta_B$ is the measured sensitivity.

| № | Sequence | Interrogation time | Readout cycles | Total time | Duty cycle | Depth (nm) | $\eta_t$ (nT/$\sqrt{\mathrm{Hz}}$) | $\eta_B$ (nT/$\sqrt{\mathrm{Hz}}$) | $E_R(\hbar)$ |
|---|---|---|---|---|---|---|---|---|---|
| 1 | XY16-128 | 0.15 ms | 904 | 0.928 ms | 16.2% | $17.3 \pm 1.0$ | 3.2 | $3.23 \pm 0.05$ | 0.85 |
| 2 | XY16-512 | 0.70 ms | 1422 | 1.889 ms | 37.1% | $26.3 \pm 0.7$ | 0.9 | $1.26 \pm 0.02$ | 0.46 |
| 3 | XY16-512 | 1.80 ms | 2025 | 3.336 ms | 54.0% | $31.7 \pm 1.1$ | 0.44 | $0.59 \pm 0.01$ | 0.18 |
| 4 | XY16-512 | 1.80 ms | 1870 | 3.247 ms | 55.4% | $49.0 \pm 1.0$ | 0.44 | $0.66 \pm 0.02$ | 0.81 |
| 5 | XY16-512 | 2.20 ms | 2030 | 3.586 ms | 61.3% | $64.3 \pm 2.0$ | 0.38 | $0.50 \pm 0.01$ | 1.1 |
| 6 | XY8-32 | 1.50 ms | 1560 | 2.623 ms | 57.2% | $80.3 \pm 3.0$ | 0.49 | $0.51 \pm 0.01$ | 2.1 |

resolution of magnetic field sensors. *Rev. Mod. Phys.* **92**, 021001 (2020).

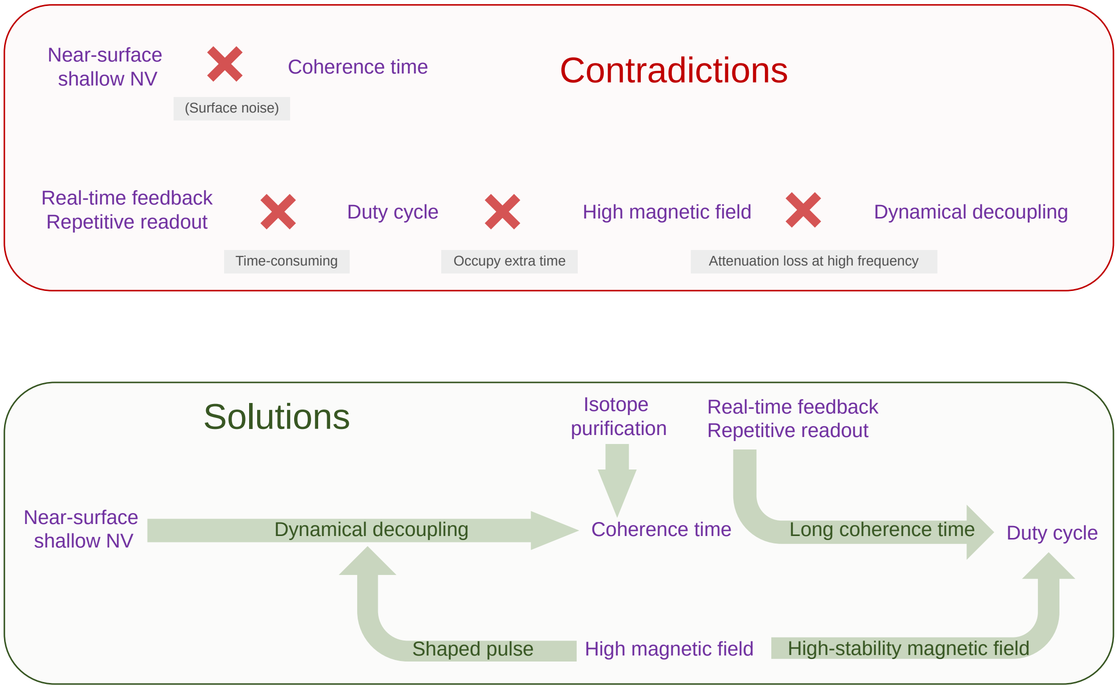


Figure S1: **Contradictions between multiple techniques and solutions.** When different technologies are combined together directly, a series of problems arise and the diagram shows our corresponding solutions.

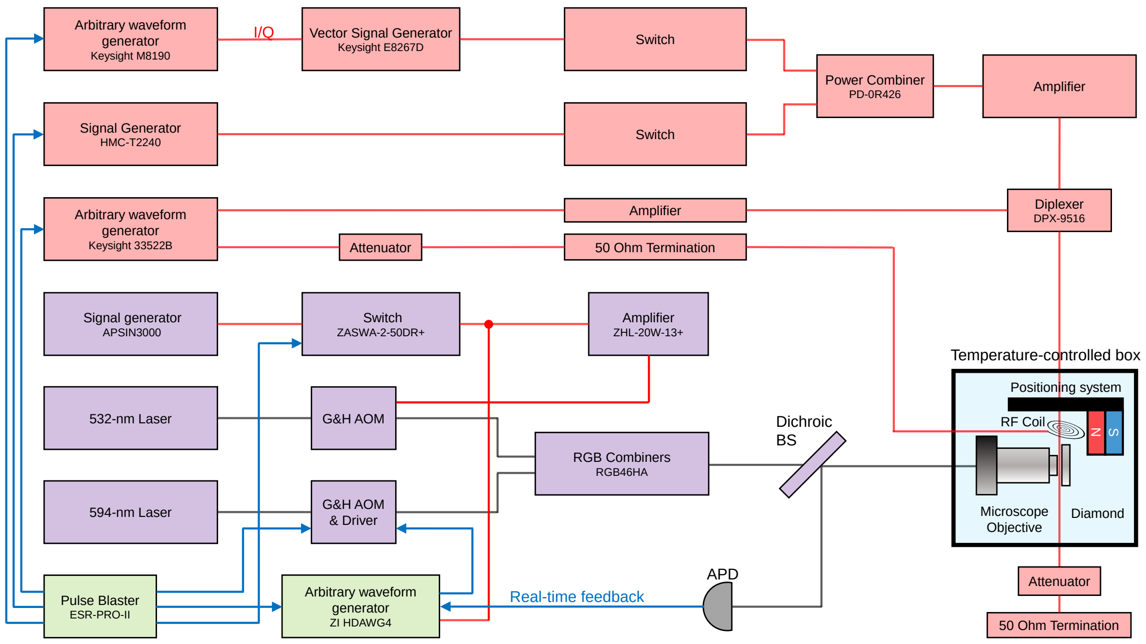


Figure S2: **Simplified experimental setup.** The setup mainly consists of microwave (MW) and radio-frequency (RF) circuits (red), optical systems (purple), synchronization and real-time feedback system (green), and a diamond platform inside a temperature-controlled box (blue). Detailed information of the experimental setup can be found in the Methods section and our previous work.

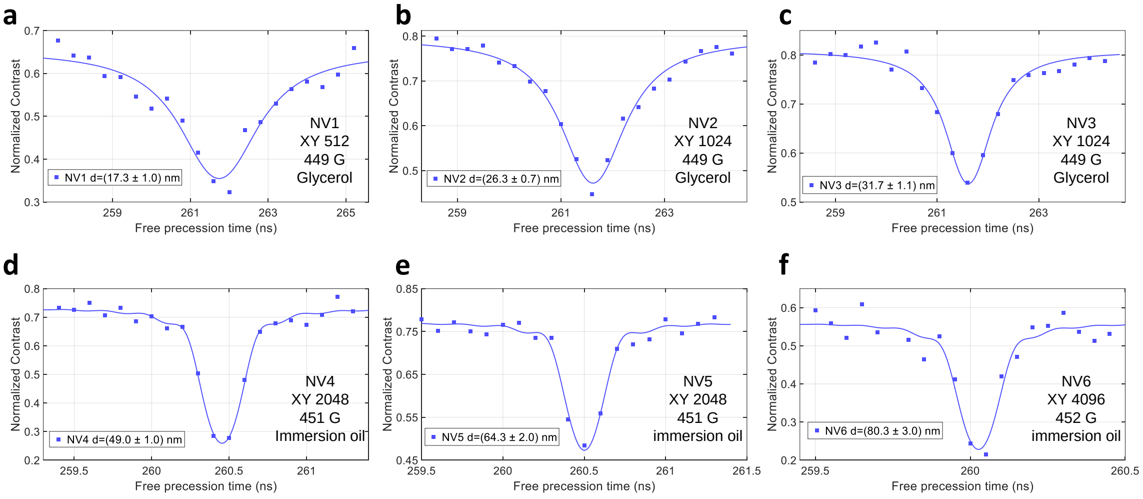


Figure S3: **NMR proton spectra detected by NV centers.** The NV number, the pulse sequence, the applied static magnetic field and the sample are given in the figures. The extracted NV depths are given in the symbol key.

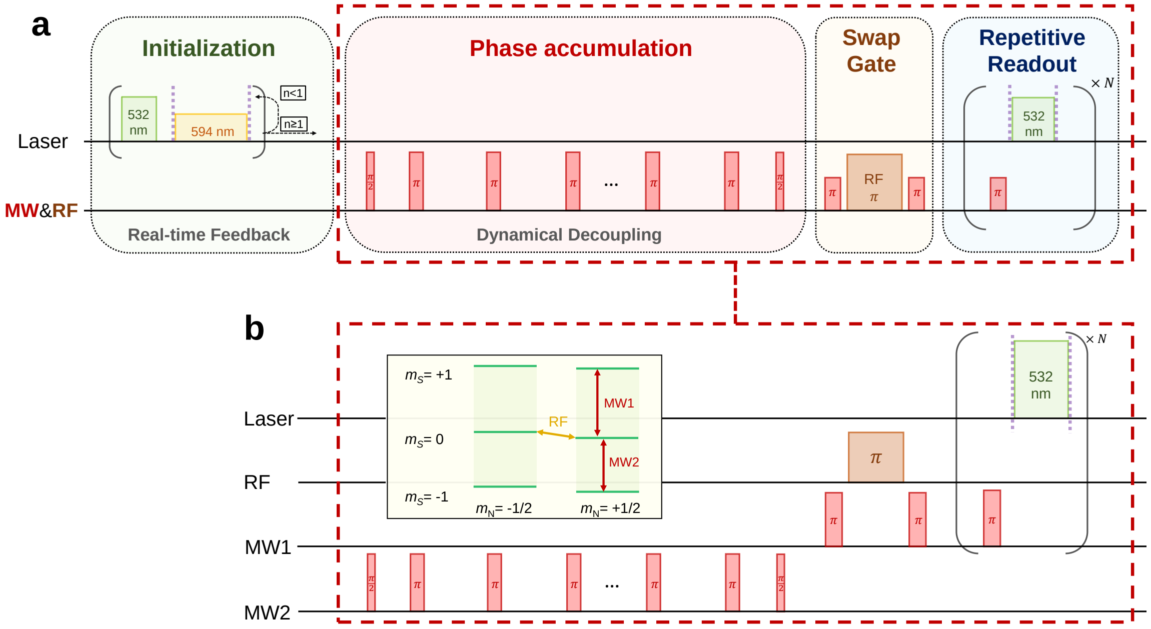


Figure S4: **Details of the interference sequecne.** (a) The interference sequecne for magnetic field measurement (Fig. 2a in the main text). (b) The details of RF and MW control for the sequence in (a) and the corresponding energy levels of our NV-$^{15}$N system for illustration. The frequencies of RF, MW1 and MW2 are 3.305 MHz, 18.6 GHz and 24.3 GHz, respectively.

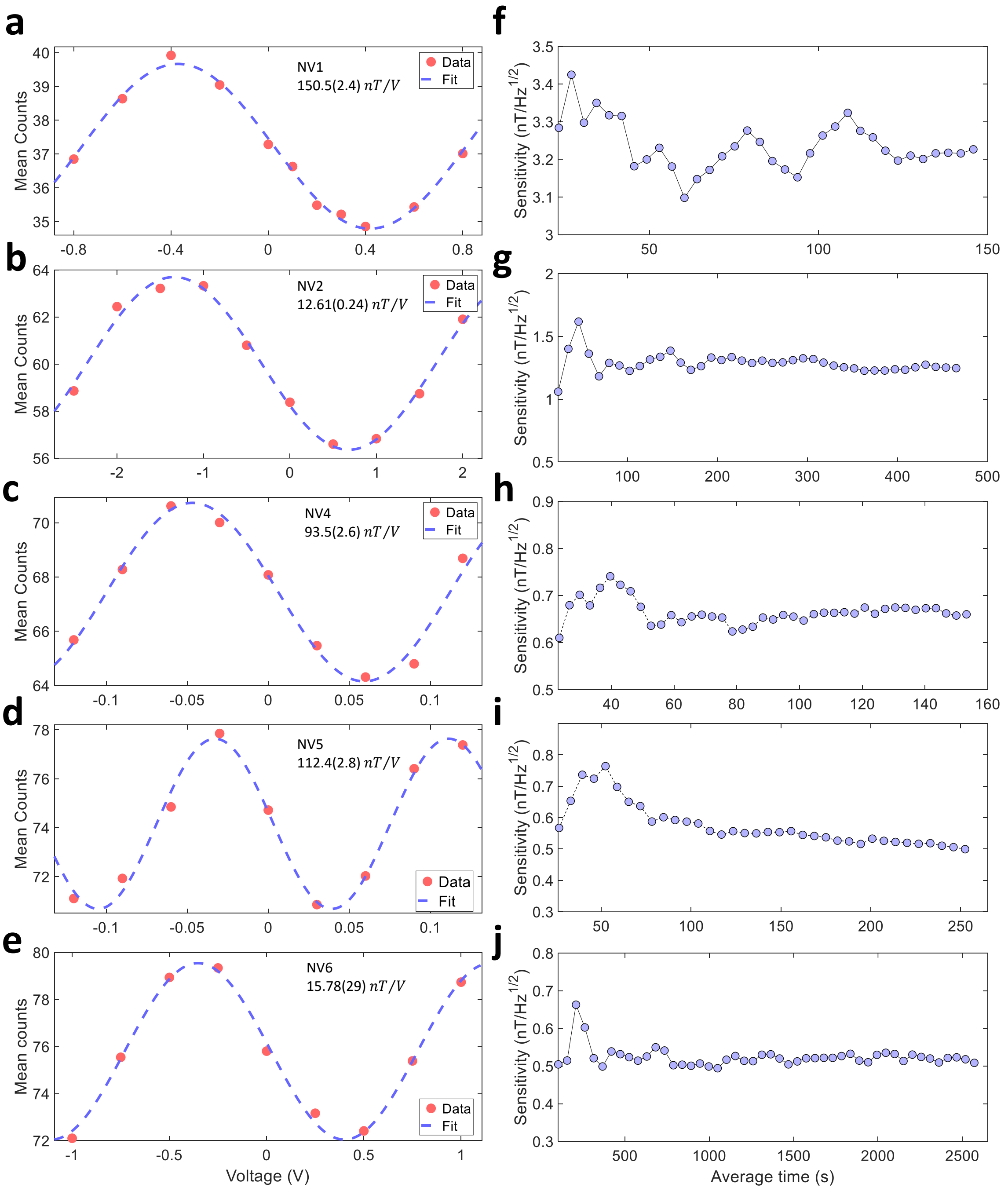


Figure S5: **Magnetic field measurement.** (a)-(e) The interference patterns for magnetic sensing. The dashed lines are given by fitting the experimental data. (f)-(j) The magnetic sensitivities for five NV centers measured as a function of the average times.

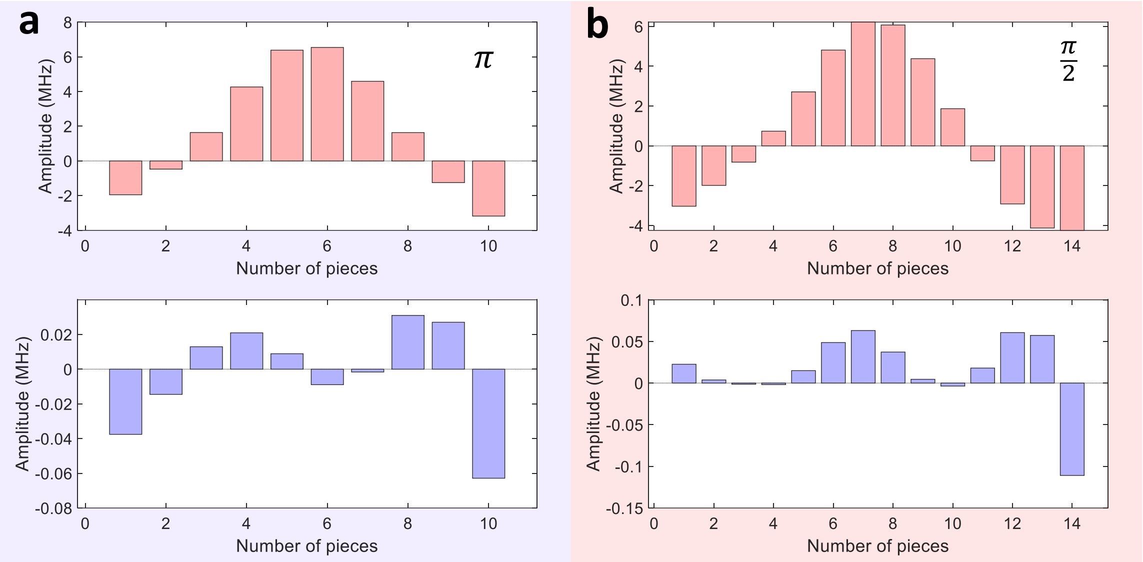


Figure S6: **Optimal control.** (a) The pulse sequence of the real (upper) and imaginary (lower) parts of the $\pi$ pulses used in dynamical decoupling sequences. The amplitude for each piece is denoted by Rabi frequency. Each piece lasts for 25 ns with 10 pieces in total. (b) The pulse sequence of the real (upper) and imaginary (lower) parts of the $\pi/2$ pulses. Each piece lasts for 25 ns with 14 pieces in total.

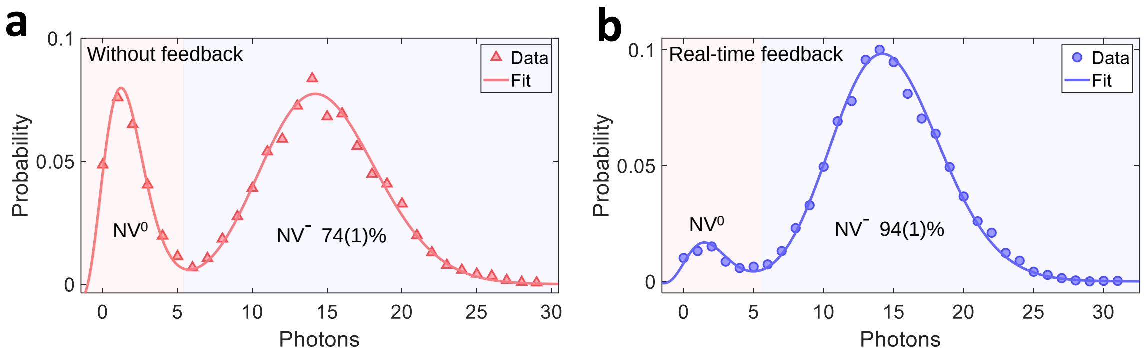


Figure S7: **Real-time feedback for NV$^-$ preparation.** (a) The distribution of two NV charge states (NV$^-$ and NV$^0$) after initialization without real-time feedback (red triangles, 74% NV$^-$). The solid lines are two-peak Poissonian fitting curves for the data points. (b) The distribution of two NV charge states after real-time-feedback initialization (blue circles, 94% NV$^-$).

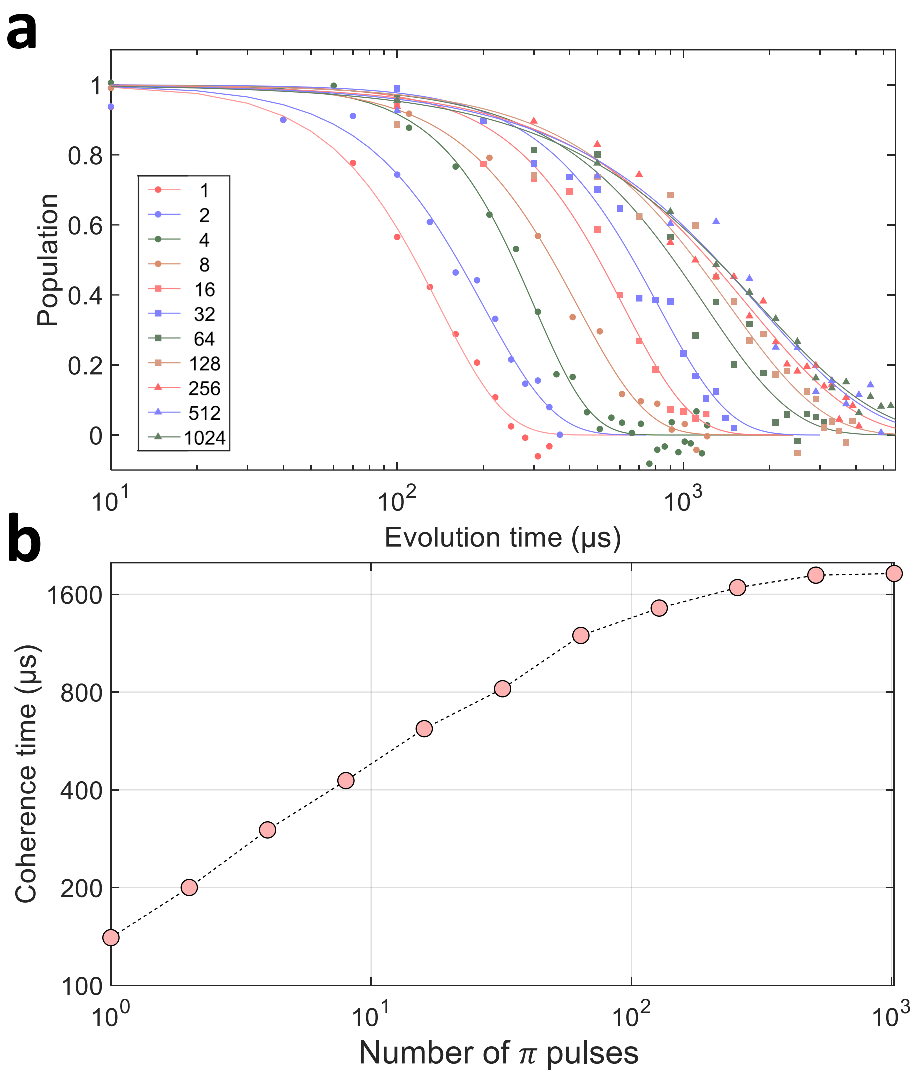


Figure S8: **Decoherence behaviors.** (a) Decoherence behaviors for the NV center with the best energy resolution under multiple dynamical decoupling sequences with different numbers of $\pi$ pulses. The solid lines are the fittings with stretched exponential functions $(\exp(-(t/T_2)^p))$. (b) Coherence times as a function of the number of $\pi$ pulses of dynamical decoupling sequences.

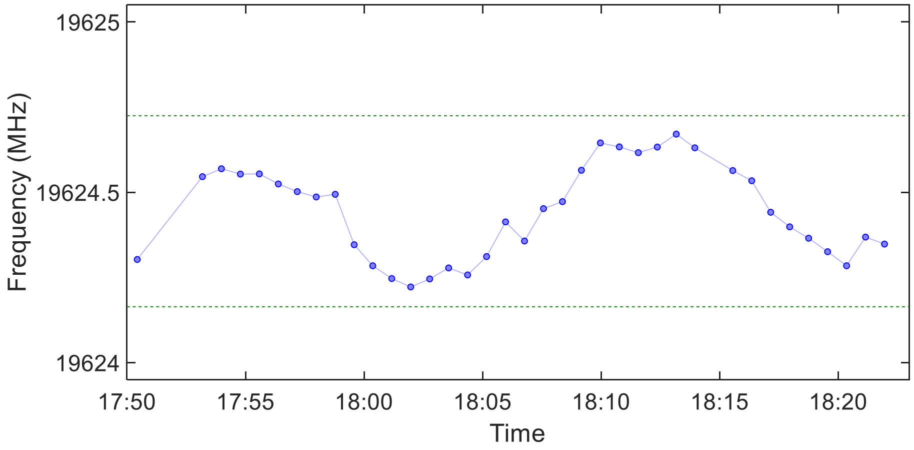


Figure S9: **Magnetic field stability.** The blue line is the shift of the resonance spectral peaks during the experiments, which shows that the bias field oscillates within 0.1 G (the green dashed lines).

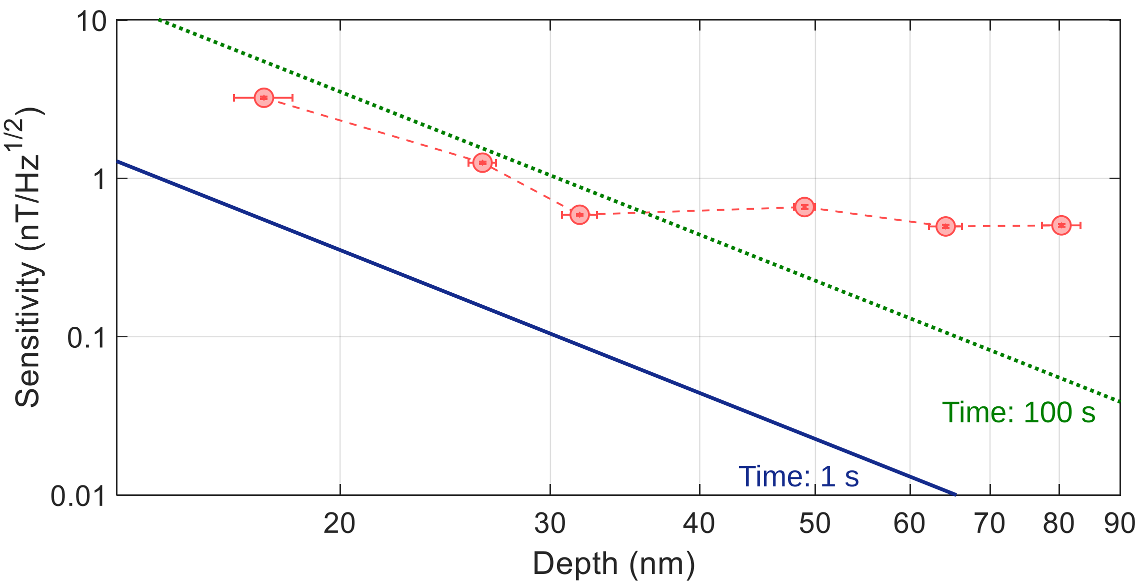


Figure S10: **Sensitivity and the signal of a single proton.** Measured sensitivities for six near-surface NV centers with different depths. The sensitivity needed for detecting a single proton (signal-to-noise ratio of 1) with 1 s (100 s) of data accumulation is calculated as a function of NV depth. For the NV center with the best energy resolution, it takes about 45 s to detect a single proton.

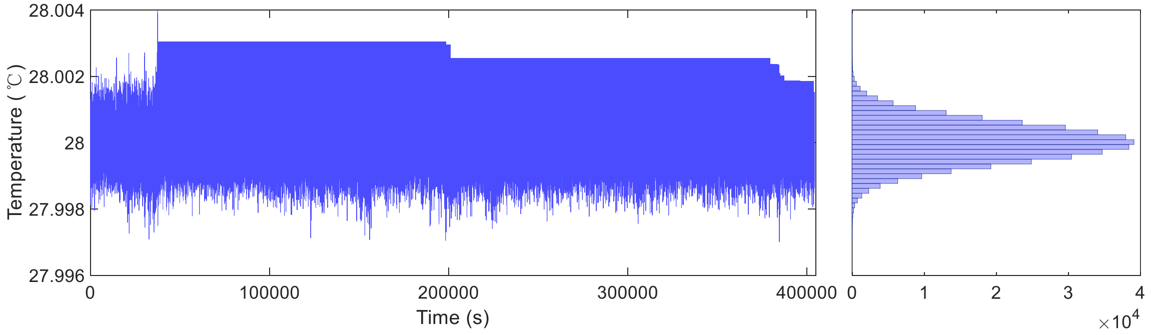


Figure S11: **Temperature stability.** Time trace of the temperature inside the copper box. The fluctuation is 0.6 mK (one standard deviation).
